# Early and late mortality following unscheduled admissions for severe liver disease across England and Wales

**DOI:** 10.1111/apt.15232

**Published:** 2019-04-11

**Authors:** Stephen E. Roberts, Ann John, Jonathan Brown, Duncan J. Napier, Ronan A. Lyons, John G. Williams

**Affiliations:** ^1^ Medical School Swansea University Swansea UK; ^2^ Health Data Research UK Swansea University Swansea UK; ^3^ Department of Gastroenterology Gloucestershire Royal Hospital Gloucester UK; ^4^ Department of Gastroenterology Royal United Hospital Bath UK

## Abstract

**Background:**

There is a known shortfall in hepatology service resources across England and Wales.

**Aim:**

To investigate early and late mortality following unscheduled admissions for severe liver disease, overall and by cause of death, and to determine how mortality is related to admissions to transplant centres, transplant surgery, hospital size, consultant specialty, patient socio‐demographics, seasonal and geographical factors.

**Methods:**

Cohorts of people with a first unscheduled admission for severe liver disease across England and Wales from 2004, based on record linkage of national inpatient and mortality data.

**Findings:**

Mortality for alcoholic liver disease and hepatic failure was 23.4% and 35.4% respectively at 60 days and 61.8% and 57.1% at 5 years. Standardised mortality ratios (SMRs) were extremely high at 60 days (184 and 117 respectively) and remained highly increased at 5 years (16.7 and 6.3). Mortality at 5 years was most elevated from liver disease, viral hepatitis and varices. The 60‐day mortality was significantly lower for patients seen by consultant hepatologists and gastroenterologists. Both early and late mortality were significantly reduced for patients admitted to transplant centres or larger hospitals, who received a liver transplant, or were resident in London. Early mortality was significantly higher for patients admitted in winter and autumn, while elevated mortality among the most vs least deprived quintile increased with longer follow‐up.

**Conclusions:**

The study shows a very poor prognosis for people with unscheduled hospitalisation for severe liver disease. The findings suggest that access to specialist expertise and services improves survival, both in the short and long term.

## 1 INTRODUCTION

There is a known shortfall in specialist liver services resources across the UK,^1^, ^2^, ^3^ which has led to the establishment of a Lancet Commission on liver disease.^2^, ^4^, ^5^, ^6^, ^7^ A number of other studies have reported on late mortality following hospitalisation for severe liver disease,^8^, ^9^, ^10^, ^11^, ^12^, ^13^, ^14^, ^15^, ^16^, ^17^, ^18^, ^19^, ^20^, ^21^, ^22^ and how it varies according to the cause of death,^9^, ^14^, ^20^, ^21^ with much of the evidence from Scandinavia.^9^, ^12^, ^15^, ^16^, ^19^, ^21^, ^22^ Little has been reported on how early and late mortality are associated with possibly important service and socio‐demographic factors.

The main objectives of this study were, firstly, to establish early and late mortality following unscheduled admission for severe liver disease across England and Wales, both overall and by cause of death. The second main objective was to establish how early and late mortality are associated with admissions to transplant centres, liver transplantation, the hospital size, consultant specialty, patient socio‐demographics, seasonal factors and the geographical region of England and Wales.

To provide confirmatory evidence, the study was based on two independently run UK National Health Services for which similar but separate data have been collected across England and Wales. The main study hypotheses were firstly, that mortality for severe liver disease would be very high, with greatly increased mortality from infections, liver cancer, accidents and suicide as well as from liver disease itself. Further hypotheses were, that mortality would be greatly reduced following liver transplantation; that early mortality would be improved for patients managed by trained hepatologists or gastroenterologists rather than by other specialists and by admission to specialist centres; and that late mortality would be worse for patients with the highest levels of social deprivation.

## 2 MATERIALS AND METHODS

The study was based on retrospective cohorts of people admitted unscheduled for severe liver disease across England and Wales. The first cohort, to investigate early mortality, included each person's first admission for severe liver disease from the start of the study in January 2004 to October 2012 with 60‐day follow‐up to the end of 2012. The second cohort, to investigate late mortality, included each person's first admission for severe liver disease from January 2004 to the end of 2007 with 5‐year follow‐up to the end of 2012.

The study cohorts were based on national administrative inpatient data, Hospital Episode Statistics for England (population 53.5 million in 2012) and the corresponding Patient Episode Database for Wales (population 3.07 million). The inpatient data were linked systematically to mortality data from the Office for National Statistics and the Welsh Demographic Service to identify deaths that occurred while in hospital or after discharge from hospital. The data were compiled and accessed through the Secure Anonymised Information Linkage (SAIL) databank.^23^, ^24^ The ascertainment of mortality has been validated as > 98% accurate and the record linkage methodology, based on a unique anonymised, encrypted linking field for each patient, as > 99.8% accurate.^23^

Severe liver disease was defined as alcoholic liver disease (International Classification of Diseases, ICD, 10th revision code, K70) or hepatic failure (K72) when based on the principal diagnosis on the discharge episode. Alcoholic liver disease was also differentiated according to the three aetiologies, alcoholic hepatitis (K70.1), alcoholic liver cirrhosis (K70.3) and alcoholic hepatic failure (K70.4).

### 2.1 Study exposure factors

Mortality was compared according to whether patients were admitted to one of the six hospitals in England in which liver transplant centres were located, in Birmingham, Cambridge, Leeds, London (2 centres) and Newcastle.^1^ For patients admitted to transplant centres, we also assessed mortality according to whether or not they were local patients, defined as resident in the same local authorities in which the transplant centres were located. We also assessed mortality according to whether patients received a liver transplant (OPCS Classification of Interventions and Procedures, OPCS‐4 code J01) in their index cohort admission.

The size of the admitting hospital was categorised by the total number of beds in five bands from < 400, 400‐599, 600‐799, 800‐999 to 1000+ beds). Consultant specialty was based, firstly, on whether or not the patients were seen by a hepatologist or gastroenterologist and compared with all other specialties (recorded during either the first or last episodes of the admission). Secondly, on whether, the patients were seen by a critical care specialist, compared with all other specialties. The season of admission was assessed by comparing winter months (December to February) with autumn (September to November), summer (June to August) and spring (March to May) as the reference category.

Social deprivation was measured using the widely used English Indices of Multiple Deprivation (IMD) for England,^25^ and the similar Welsh Index of Multiple Deprivation (WIMD) for Wales.^26^ The total IMD and WIMD deprivation scores for geographical Lower Super Output Areas (LSOAs) (average LSOA population = 1640 in England and 1580 in Wales) were ranked and categorised into quintiles (I = least deprived and V = most deprived quintile).

The regions of England and Wales were based on the patients’ recorded residence and the conventional Government Office Regional classification which includes 10 regional categories.^27^ Namely, these are London, South East of England, South West of England, East of England, West Midlands, East Midlands, Yorkshire and Humberside, North West of England, North East of England, and Wales.

### 2.2 Outcome measures

The main outcome measures were ‘early mortality’, defined as all deaths within 60 days of admission and ‘late mortality’, all deaths within 5 years of admission. Secondary outcome measures were standardised mortality ratios (SMRs) at 60 days and 5‐year follow‐up and also relative survival at monthly intervals up to 5 years. They were used to compare mortality in the cohorts of patients hospitalised for severe liver disease with those in the corresponding general populations of England and Wales. Cause‐specific SMRs at 5‐year follow‐up were based on the underlying causes of death on death certificates.

### 2.3 Methods of analysis

Age and sex adjusted SMRs were calculated using the indirect method, by applying age and sex specific mortality in the general adult resident populations of England of Wales to obtain the expected mortality and by then comparing observed and expected mortality. The age groups used were 18‐19 years, 20‐24, quinquennially up to 80‐84 and then 85+ years. Relative survival was calculated as a ratio to compare the observed survival in the cohorts of patients hospitalised for severe liver disease with that expected in the corresponding (age‐ and sex‐matched) general populations of England and Wales.

Multivariate logistic regression models were used to assess associations between the study exposure factors and subsequent mortality. In the models, mortality was adjusted for patient age (in 5‐year groups from 35 to 85+ years, with < 35 years as the reference category), sex and 10 major patient co‐morbidities (ischaemic heart disease, other cardiovascular diseases, cerebrovascular disease, other circulatory diseases, malignancies, chronic obstructive pulmonary disease (COPD), asthma, diabetes, renal failure and dementia; ICD‐10 codes are listed for each co‐morbidity in the Appendix A). The co‐morbidities were based on a diagnosis recorded in any position on the patients' current inpatient record or on previous inpatient records during the preceding 5 years. To eliminate any possible biases in the determination of patient co‐morbidities from inpatient admissions alone, adjustment was also made for patients with no previous admissions during the preceding 5 years. Patient sex was missing in < 0.01% of cases (5 of 73 123), postcode‐based social deprivation in 2.3% (1668), consultant specialty in < 0.01% (15), residential local authority in 4.1% of patients admitted to liver transplant centres (110 of 2653) and hospital size (coded only at trust level; 18.5%, 13 531). Missing data were excluded from the analyses involving the respective factors. There were no missing data for patient age, residential region, admissions to transplant centres, cause of death and day of admission or death.

Additionally, through record linkage of the inpatient data, we identified subsequent emergency admissions for severe liver disease in the study cohort patients and compared survival according to the numbers of subsequent admissions. When assessing trends in early mortality for alcoholic liver disease and for hepatic failure, annual mortality rates were standardised using the direct method and the total populations of patients admitted as the standard populations. Logistic regression modelling was used to obtain mean annual reductions over time in the age and sex adjusted early mortality rates for alcoholic liver disease and hepatic failure. Other methods used were unpaired *t* tests to compare patient ages, chi‐squared tests to compare patient co‐morbidities and socio‐demographics and the Mann‐Whitney test to compare lengths of inpatient stay. Statistical significance was measured at the conventional 5% level.

## 3 RESULTS

In the first study cohort with 60‐day follow‐up to investigate early mortality, there were a total of 73 123 patients hospitalised with severe liver disease; mean age = 52.8 years (SD = 12.7) and 35.3% were female. In the second cohort with 5‐year follow‐up to establish late mortality, there were a total of 33 726 patients; mean age = 52.5 years (SD = 12.2) and 35.0% were female.

### 3.1 Early and late mortality

For England and Wales combined, early 60‐day mortality (cohort 1) for alcoholic liver disease and hepatic failure was 23.4% and 35.4% respectively (Table 1). Late 5‐year mortality was respectively 61.8% and 57.1% (cohort 2). When comparing mortality with the corresponding general resident populations of England and Wales, SMRs were higher throughout follow‐up for alcoholic liver disease than for hepatic failure, they were extremely high at 60‐day follow‐up (184 for alcoholic liver disease and 117 for hepatic failure, compared with 1.0 in the general population) and remained highly elevated in the longer term (16.7 for alcoholic liver disease and 6.3 for hepatic failure at 5 years).

**Table 1:** Early and late mortality rates and standardised mortality ratios (SMRs) following unscheduled admissions for severe liver disease in England and Wales overall and according to aetiology

	Early mortality cohort (60‐d follow‐up)					Late mortality cohort (5‐y follow‐up)				
	No. of admissions	No. of deaths	Mortality (%)	SMR	(95% CI)	No. of admissions	No. of deaths	Mortality (%)	SMR	(95% CI)
England and Wales										
Severe liver disease	73 123	18 194	24.9	167.2	(176.0, 181.0)	33 726	20 685	62.8	14.3	(14.1, 14.5)
Alcoholic liver disease	64 145	15 014	23.4	184.0	(181.1, 186.9)	30 057	18 590	61.8	16.7	(16.5, 16.9)
Alcoholic hepatitis	10 817	1666	15.4	198.8	(189.3, 208.4)	4318	1994	46.2	20.1	(19.3, 21.0)
Alcoholic liver cirrhosis	16 764	4426	26.4	158.3	(153.7, 163.0)	7118	4917	69.1	14.7	(14.3, 15.2)
Alcoholic liver failure	7155	3267	45.7	356.4	(344.3, 368.7)	2592	2006	77.4	20.2	(19.4, 21.1)
Hepatic failure	8978	3180	35.4	116.8	(112.8, 121.0)	3669	2095	57.1	6.3	(6.0, 6.6)
England										
Severe liver disease	68 219	17 014	24.9	168.5	(165.9, 171.0)	31 389	19 217	61.2	14.4	(14.2, 14.6)
Alcoholic liver disease	59 765	14 032	23.5	185.8	(182.7, 188.9)	27 957	17 269	61.8	16.8	(16.6, 17.1)
Alcoholic hepatitis	10 296	1576	15.3	197.5	(187.9, 207.4)	4100	1895	46.2	20.1	(19.2, 21.1)
Alcoholic liver cirrhosis	15 627	4116	26.3	159.7	(154.9, 164.6)	6637	4566	68.8	14.7	(14.3, 15.2)
Alcoholic liver failure	6734	3074	45.6	353.2	(340.8, 365.8)	2457	1893	77.0	20.2	(19.3, 21.1)
Hepatic failure	8454	2982	35.3	117.1	(113.0, 121.4)	3432	1948	56.8	6.3	(6.0, 6.6)
Wales										
Severe liver disease	4904	1180	24.1	155.8	(147.5, 164.4)	2337	1468	62.8	14.0	(13.3, 14.7)
Alcoholic liver disease	4380	982	22.4	161.7	(151.8, 172.0)	2100	1321	62.9	16.0	(15.1, 16.9)
Alcoholic hepatitis	521	90	17.3	224.2	(180.4, 273.2)	218	99	45.4	20.2	(16.4, 24.4)
Alcoholic liver cirrhosis	1137	310	27.3	141.6	(126.2, 157.8)	481	351	73.0	13.9	(12.5, 15.4)
Alcoholic liver failure	381	193	50.7	416.5	(359.8, 477.3)	135	113	83.7	20.7	(17.1, 24.7)
Hepatic failure	524	198	37.8	112.7	(95.7, 128.9)	237	147	62.0	6.6	(5.6, 7.7)

**Figure 1:**
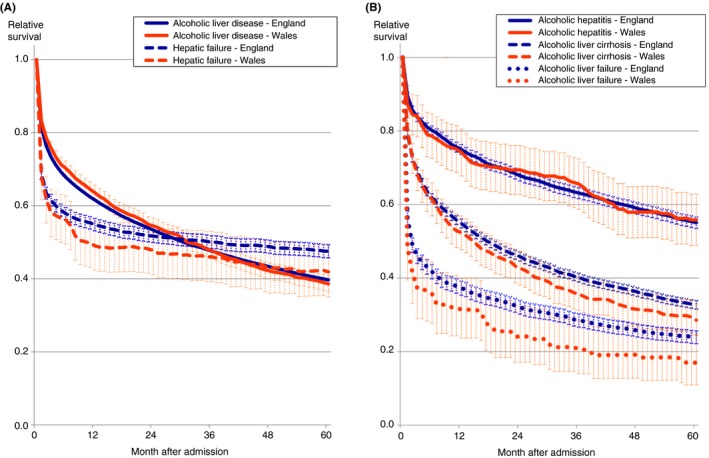
Relative survival up to 5 y following unscheduled admissions for severe liver disease in England and Wales, compared with the corresponding age‐ and sex‐matched general populations. A, Alcoholic liver disease and hepatic failure. B, Alcoholic hepatitis, alcoholic liver cirrhosis and alcoholic liver failure. Shaded areas represent 95% CIs

Early survival was substantially worse following admission with hepatic failure than for alcoholic liver disease, approximately equal by 3‐year follow‐up, but over longer term follow‐up, it deteriorated much more severely for alcoholic liver disease (Figure 1A). Of the three main categories of alcoholic liver disease, prognosis was worst for alcoholic liver failure, followed by alcoholic liver cirrhosis, but comparatively better for alcoholic hepatitis (Figure 1B).

**Figure 2:**
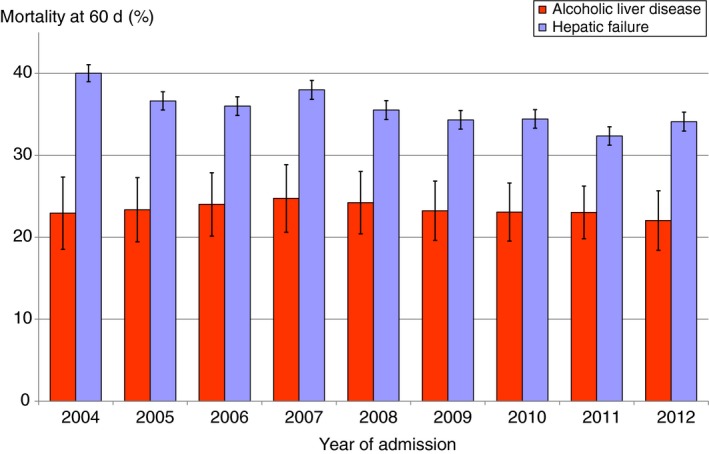
Trends in early (60‐d) mortality for alcoholic liver disease and hepatic failure in England and Wales. Mortality rates are standardised for age group and sex. Vertical bars represent 95% CIs

Early 60‐day mortality fell significantly over time during the study period, although this was greater for hepatic failure than for alcoholic liver disease (Figure 2). The mean annual reduction was 3.4% (95% CI = 1.8%‐5.1%) for hepatic failure and 0.7% (0.0%‐1.4%) for alcoholic liver disease.

### 3.2 Cause‐specific mortality

Table 2 shows cause‐specific mortality (SMRs) for late mortality (5‐year follow‐up) separately for patients admitted with alcoholic liver disease or with hepatic failure. At 5 years, liver disease accounted for 72.2% of all deaths among those originally admitted with alcoholic liver disease and 51.0% of those admitted with hepatic failure (Table 2). Late mortality was extremely increased (SMR > 100) from most of the major causes of death from liver disease and also from viral hepatitis and varices (although the SMR for varices was lower at 71.7 in patients admitted for hepatic failure).

**Table 2:** Underlying causes of death and corresponding SMRs at 5 y following unscheduled admissions for alcoholic liver disease and hepatic failure in England and Wales

Underlying cause of death	ICD‐10 codes	Alcoholic liver disease				Hepatic failure			
		No. of deaths	(% of all deaths)	SMR	(95% CI)	No. of deaths	(% of all deaths)	SMR	(95% CI)
Gastrointestinal diseases	K00‐K93	13 886	(74.7)	184.3	(181.2, 187.4)	1164	(55.6)	66.4	(62.6, 70.2)
Liver disease	K70‐K77	13 429	(72.2)	333.2	(327.6, 338.8)	1068	(51.0)	239.6	(225.4, 254.2)
Alcoholic liver disease	K70	10 861	(58.4)	378.3	(371.2, 385.4)	497	(23.7)	184.5	(168.7, 201.1)
Hepatic failure	K72	170	(0.9)	271.3	(232.1, 313.6)	117	(5.6)	1068.3	(883.5, 1270.6)
Other liver disease	K71, K73‐K77	2398	(12.9)	218.6	(210.0, 227.5)	454	(21.7)	274.3	(249.7, 300.1)
Fibrosis & cirrhosis of liver	K74	1651	(8.9)	247.2	(235.5, 259.3)	328	(15.7)	336.2	(300.8, 373.6)
Non‐alcoholic hepatic steatosis	K76.0	71	(0.4)	45.9	(35.9, 57.3)	29	(1.4)	169.2	(113.2, 236.4)
Portal hypertension	K76.6	14	(0.1)	317.2	(184.3, 485.9)	*	*	213.8	(20.2, 612.8)
Hepatorenal syndrome	K76.7	89	(0.5)	492.1	(395.2, 599.6)	*	*	173.3	(33.8, 358.4)
Other gastrointestinal diseases	K00‐K67, K80‐K93	457	(2.5)	13.0	(12.0, 14.3)	96	(4.6)	7.3	(5.9, 8.8)
Oesophageal and gastric varices	I85, I86.4	24	(0.1)	130.2	(83.3, 187.4)	*	*	71.7	(6.8, 205.6)
Oesophageal ulcer	K22.1	20	(0.1)	41.0	(25.0, 60.9)	*	*	9.6	(0.0, 37.8)
Perforated peptic ulcer & peritonitis	K25‐K27, K65	170	(0.9)	21.1	(18.6, 25.1)	16	(0.8)	6.7	(3.8, 11.4)
Herniae	K40‐K43, K45, K46	20	(0.1)	14.9	(9.1, 22.1)	*	*	3.8	(0.4, 11.0)
Intestinal obstruction	K56	12	(0.1)	4.3	(2.2, 7.2)	*	*	0.8	(0.0, 3.0)
Diverticular disease	K57	16	(0.1)	6.7	(3.8, 10.4)	*	*	3.1	(0.8, 7.0)
Acute pancreatitis	K85	44	(0.2)	15.7	(11.4, 20.7)	10	(0.5)	14.8	(7.1, 25.4)
Chronic pancreatitis	K86.0, K86.1	26	(0.1)	35.7	(23.3, 50.8)	*	*	12.5	(0.0, 48.8)
Cancers	C00‐C96	852	(4.6)	2.1	(2.0, 2.3)	241	(11.5)	2.5	(2.2, 2.8)
Liver cancer	C22	316	(1.7)	30.8	(27.5, 34.3)	71	(3.4)	32.6	(25.5, 40.7)
Other gastrointestinal cancers	C15‐C21, C23‐C26	124	(0.7)	1.1	(1.0, 1.4)	52	(2.5)	2.0	(1.5, 2.6)
Oesophageal cancer	C15	46	(0.2)	1.9	(1.4, 2.5)	*	*	0.4	(0.0, 1.2)
Gastric cancer	C16	6	(0.0)	0.5	(0.2, 0.9)	*	*	0.3	(0.0, 1.1)
Colorectal cancer	C18‐C20	36	(0.2)	1.7	(1.2, 2.3)	16	(0.8)	3.2	(1.9, 2.5) 5.0)
Pancreatic cancer	C25	23	(0.1)	1.1	(0.7, 1.6)	27	(1.3)	5.5	(3.6, 7.7)
Other cancers	C00‐C14, C30‐C97	412	(2.2)	1.4	(1.3, 1.6)	118	(5.6)	1.7	(1.4, 2.0)
Mouth and throat cancer	C00‐C14, C32	88	(0.5)	7.9	(6.3, 9.6)	*	*	2.8	(0.9, 5.8)
Lung cancer	C33, C34	116	(0.6)	1.2	(1.0, 1.4)	15	(0.7)	0.7	(0.4, 1.1)
Breast cancer	C50	21	(0.1)	1.0	(0.6, 1.4)	26	(1.2)	4.3	(2.8, 6.1)
Prostate cancer	C61	17	(0.1)	0.7	(0.4, 1.0)	10	(0.5)	1.3	(0.6, 2.2)
Lymphomas	C81‐C96	15	(0.1)	0.5	(0.3, 0.8)	16	(0.8)	2.1	(1.2, 3.3)
Infectious diseases	A00‐B99	314	(1.7)	23.8	(21.3, 26.5)	126	(6.0)	28.8	(24.0, 34.0)
Viral hepatitis	B15‐B19	160	(0.9)	123.6	(105.2, 143.5)	93	(4.4)	685.5	(533.3, 831.9)
Septicaemia	A40, A41	92	(0.5)	23.8	(19.2, 28.9)	21	(1.0)	14.5	(9.1, 21.4)
Circulatory diseases	I00‐I99	1333	(7.2)	4.0	(3.8, 4.2)	239	(11.4)	2.0	(1.7, 2.2)
Ischaemic heart disease	I20‐I25	517	(2.8)	2.8	(2.5, 3.0)	109	(5.2)	1.9	(1.6, 2.3)
Stroke	I61‐I64	271	(1.5)	5.8	(5.1, 6.5)	26	(1.2)	1.1	(0.7, 1.6)
Respiratory diseases	J00‐J99	614	(3.3)	5.2	(4.6, 5.4)	62	(3.0)	1.2	(0.9, 1.5)
Pneumonia	J12‐J18	216	(1.2)	6.1	(5.2, 6.7)	32	(1.5)	1.6	(1.1, 2.1)
COPD	J40‐J44	190	(1.0)	3.5	(3.0, 4.1)	11	(0.5)	0.6	(0.3, 1.0)
Mental and behavioural disorders	F00‐F99	272	(1.5)	13.7	(12.1, 15.4)	19	(0.9)	1.5	(0.9, 2.3)
Due to alcohol use	F10	210	(1.1)	54.8	(47.6, 62.4)	13	(0.6)	35.5	(18.9, 57.5)
Due to drug use	F11‐F19	47	(0.3)	15.6	(11.5, 20.4)	*	*	7.3	(0.7, 21.0)
Injury and poisoning	V01‐Y98	517	(2.8)	9.7	(8.9, 10.6)	52	(2.5)	5.4	(4.1, 7.0)
Accidental falls	W00‐W19	145	(0.8)	18.9	(15.9, 22.1)	8	(0.4)	3.3	(1.4, 6.1)
Transport accidents	V01‐V89	25	(0.1)	3.2	(2.1, 4.6)	0	(0.0)	0	
Accidental poisoning	X40‐X49	117	(0.6)	21.2	(17.5, 25.2)	18	(0.9)	33.4	(19.8, 50.6)
Homicide	X85‐Y09	*	(0.0)	3.9	(1.0, 8.7)	0	(0.0)	0	
Suicide	X60‐X84, Y10‐Y33.8	80	(0.4)	4.0	(3.2, 4.9)	14	(0.7)	6.8	(3.7, 10.8)
All causes of death	A00‐Z99	18 590	(100)	16.7	(16.5, 16.9)	2095	(100)	(6.3)	(6.0, 6.6)

* Denotes small numbers of cases ≤ 5.

Late mortality was also very highly increased (SMRs > 10) from liver cancer, acute and chronic pancreatitis, septicaemia, infectious diseases generally, mental and behavioural disorders due to alcohol and from accidental poisoning (Table 2). Late mortality was also very highly increased (SMRs > 10) among patients admitted with alcoholic liver disease, but not with hepatic failure, from certain gastrointestinal diseases (oesophageal ulcer, perforated peptic ulcer and peritonitis and herniae), accidental falls, mental and behavioural disorders generally and due to drugs.

There was relatively little or no increased mortality from cancers overall and from the major types of cancer, with the exceptions of mouth and throat cancer in people admitted with alcoholic liver disease (SMR = 7.9) and pancreatic cancer in those admitted with hepatic failure (SMR = 5.5; Table 2). Among patients admitted for hepatic failure, there was little or no increased late mortality from circulatory diseases generally, ischaemic heart disease, respiratory diseases generally, COPD and pneumonia.

### 3.3 Factors that may influence prognosis

**Figure 3:**
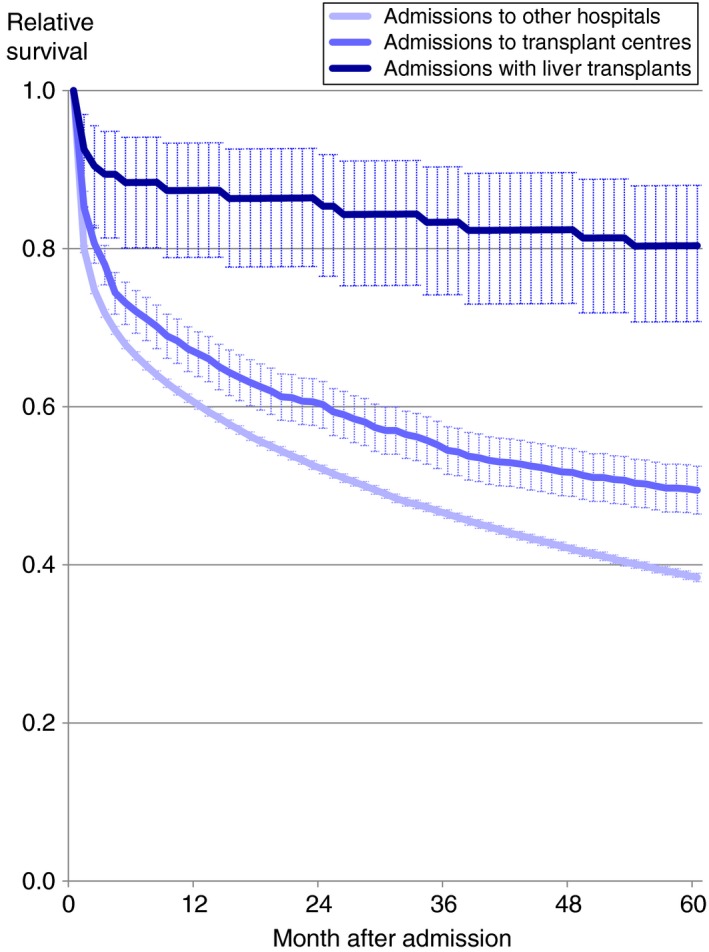
Relative survival up to 5 y following unscheduled admissions for severe liver disease in England and Wales according to liver transplant surgery, admissions to transplant centres and to all other hospitals**. **Shaded areas represent 95% CIs

Early 60‐day mortality but not late 5‐year mortality was significantly lower among patients seen by hepatologists or gastroenterologists (by 22.3% compared with other specialties; Table 3). Both early and late mortality were significantly much lower among patients admitted to specialist transplant centres (by 26.5% and 38.6% respectively), who received liver transplants (by 4‐ and 7‐fold respectively; Table 3; Figure 3) and who were resident in London compared with all other major regions of England and Wales. Both early and late mortality were also significantly reduced among patients admitted to larger compared with smaller hospitals, although this pattern was stronger for early mortality.

**Table 3:** Early and late mortality following unscheduled admissions for severe liver disease in England and Wales in relation to consultant specialty, hospital size, admissions to liver transplant centres, geographical residential region, season and socio‐demographics

	Early mortality (60‐d follow‐up)				Late mortality (5‐y follow‐up)			
	No. of admissions	Mortality rate (%)	Odds ratio	(95% CI)	No. of admissions	Mortality rate (%)	Odds ratio	(95% CI)
Specialty								
Gastro/ hepatology	25 978	22.4	Ref		8892	61.2	Ref	
Other specialties	47 130	26.3	1.223	(1.177, 1.270)	24 822	61.4	0.988	(0.938, 1.041)
Hospital size (beds)								
<400	11 310	25.9	Ref		4438	62.4	Ref	
400‐599	22 450	25.6	1.001	(0.947, 1.058)	10 182	62.4	0.993	(0.920, 1.071)
600‐799	13 381	25.0	0.996	(0.937, 1.060)	5983	61.0	0.964	(0.887, 1.048)
800‐999	8966	21.4	0.812	(0.756, 0.871)	3652	58.2	0.874	(0.796, 0.960)
1000+	3485	23.4	0.884	(0.804, 0.972)	1597	61.1	0.935	(0.827, 1.057)
Admissions to a liver transplant centre								
No	70 470	25.9	Ref		32 633	61.7	Ref	
Yes	2653	20.5	0.735	(0.664, 0.814)	1093	50.9	0.614	(0.541, 0.697)
Transplant centre admissions								
Local patients	1524	22.7	Ref		590	55.4		
Non‐local patients	1019	16.7	0.637	(0.509, 0.796)	440	48.4	0.693	(0.527, 0.912)
Liver transplant surgery (during index cohort admission)								
Yes	140	7.9	Ref		94	20.2	Ref	
No	72 983	24.9	4.037	(2.151, 7.575)	33 632	61.4	7.218	(4.293, 12.14)
Liver transplant surgery								
Local patients	15	0.0	Ref		7	14.3	Ref	
Non‐local patients	125	8.8	*	*	87	20.7	0.763	(0.041, 14.34)
Residential region								
London	9736	21.2	Ref		4591	54.9	Ref	
South East	8318	26.6	1.291	(1.212, 1.383)	3867	63.5	1.335	(1.218, 1.468)
South West	5908	25.5	1.221	(1.144, 1.324)	2677	63.2	1.276	(1.152, 1.414)
East of England	5536	24.7	1.257	(1.163, 1.350)	2614	60.8	1.213	(1.094, 1.343)
East Midlands	5006	25.7	1.284	(1.199, 1.398)	2279	62.2	1.296	(1.164, 1.443)
West Midlands	7816	27.8	1.466	(1.355, 1.549)	3597	62.6	1.398	(1.274, 1.535)
Yorkshire & Humber	7300	25.3	1.359	(1.274, 1.462)	3292	61.9	1.350	(1.227, 1.485)
North East	4682	24.2	1.300	(1.200, 1.404)	2140	61.0	1.324	(1.187, 1.477)
North West	13 811	24.3	1.333	(1.251, 1.409)	6316	62.3	1.473	(1.358, 1.597)
Wales	5010	24.0	1.236	(1.152, 1.354)	2353	62.8	1.369	(1.231, 1.522)
Season admitted								
Spring	18 455	24.0	Ref		8434	61.1	Ref	
Summer	18 977	23.8	0.994	(0.945, 1.045)	8686	60.3	0.980	(0.919, 1.045)
Autumn	18 464	25.6	1.091	(1.038, 1.148)	8280	61.9	1.040	(0.975, 1.110)
Winter	17 227	26.3	1.128	(1.072, 1.187)	8326	62.0	1.029	(0.964, 1.098)
Patient age group								
18‐35	5123	10.5	Ref		2217	35.1	Ref	
35‐44	14 414	15.6	1.702	(1.537, 1.884)	6945	51.0	1.922	(1.738, 2.124)
45‐54	21 565	21.8	2.618	(2.375, 2.885)	10 250	59.5	2.793	(2.533, 3.079)
55‐64	19 101	28.6	3.838	(3.480, 4.232)	8728	65.5	3.944	(3.565, 4.363)
65‐74	9267	36.3	5.745	(5.179, 6.372)	3988	76.9	7.003	(6.215, 7.890)
75+	3653	51.8	10.91	(9.695, 12.27)	1598	87.6	14.26	(11.97, 16.99)
Patient sex								
Male	47 317	24.6	Ref		21 904	62.2	Ref	
Female	25 801	25.5	1.063	(1.024, 1.103)	11 820	59.6	0.892	(0.850, 0.936)
Social deprivation								
I	7719	27.6	Ref		3455	62.9	Ref	
II	9884	27.1	1.002	(0.933, 1.077)	4481	62.6	1.016	(0.923, 1.119)
III	12 270	25.5	0.997	(0.931, 1.068)	5516	62.5	1.090	(0.994, 1.195)
IV	16 693	24.3	1.020	(0.956, 1.089)	7745	60.8	1.085	(0.995, 1.183)
V	24 889	23.7	1.092	(1.026, 1.162)	11 711	61.6	1.210	(1.114, 1.315)
Specialty								
Critical care medicine	749	76.1	Ref		212	89.2	Ref	
Other specialties	72 374	24.4	0.111	(0.093, 0.133)	33 514	61.2	0.197	(0.127, 0.306)

Ref = comparison reference category.

* Denotes zero mortality so that a logistic regression model cannot be applied.

Early but not late mortality was significantly higher among patients admitted during winter and autumn compared with other months (Table 3). Among women compared with men, early mortality was significantly increased (by 6.3%) but late mortality was reduced (by 10.8%). Mortality was also significantly worse among patients with the highest levels of social deprivation compared with the lowest (quintile V vs I) and this differential increased from early mortality (9.2%) to late mortality (21.0%). There were no other significant differences between deprivation quintiles.

Both early and late mortality were greatly increased among patients seen by critical care consultants, by 9‐fold and 5‐fold respectively (Table 3). Compared with all other patients admitted acutely with severe liver disease, those seen by critical care specialists were less often: diagnosed with alcoholic liver disease (73.8% vs 87.9%; *P* < 0.001), male (59.4% vs 64.8%; *P* = 0.002), and (non‐significantly) socially deprived (54.6% vs 58.3% in the two most deprived quintiles; *P* = 0.067) but were of similar ages (mean = 52.2 vs 52.8 years; *P* = 0.201). They also had longer inpatient stays (median = 14.0 vs 9.0 days; *P* < 0.001).

### 3.4 Patients admitted to transplant centres

Among patients admitted to transplant centres, both early and late mortality were substantially higher among those resident locally, compared with patients admitted from other local authorities (Table 3). ‘Local’ compared with ‘non‐local’ patients, were of similar age (52.6 vs 53.0 years respectively; *P* = 0.425) and gender (33.7% vs 35.0% women; *P* = 0.496), but had higher levels of social deprivation (48.4% from the most deprived quintile V vs 19.7%; *P* < 0.001). Of the major co‐morbidities, local patients had higher levels of COPD (11.3% vs 6.1%; *P* < 0.001), lower levels of renal failure (21.5% vs 27.8%; *P* = 0.006), cancers (8.4% vs 15.5%; *P* < 0.001) and diabetes (16.7% vs 22.8%; *P* < 0.001), but similar levels of stroke (5.0% vs 4.0%; *P* = 0.256) and ischaemic heart disease co‐morbidities (10.4% vs 8.7%; *P* = 0.157). When compared with locally resident patients, the diagnosis for patients transferred to transplant centres from elsewhere was more often for hepatic failure (31.1% of cases vs 17.9%; *P* < 0.001), a diagnosis that has a better longer term prognosis than alcoholic liver disease, and less often for alcoholic liver failure (3.9% vs 9.3%; *P* < 0.001) which has the worst prognosis of the subtypes.

The 140 patients who received liver transplants were mostly not resident locally (89%), were slightly but not significantly younger (51.8% vs 52.8%; *P* = 0.103) and more affluent (14.7% vs 34.9% in quintile V; *P* < 0.001) than other patients with severe liver disease. On admission, they also had higher levels of cancers (20.0% vs 7.3%; *P* < 0.001), diabetes (35.0% vs 15.7%; *P* < 0.001) and renal failure co‐morbidities (32.1% vs 18.7%; *P* < 0.001) but lower levels of COPD (3.6% vs 9.1%; *P* = 0.024). Of 19 deaths in the 5 years following liver transplantation, alcoholic disorders were not recorded as a cause of death in a single case.

### 3.5 Subsequent admissions for severe liver disease in the cohort patients

**Figure 4:**
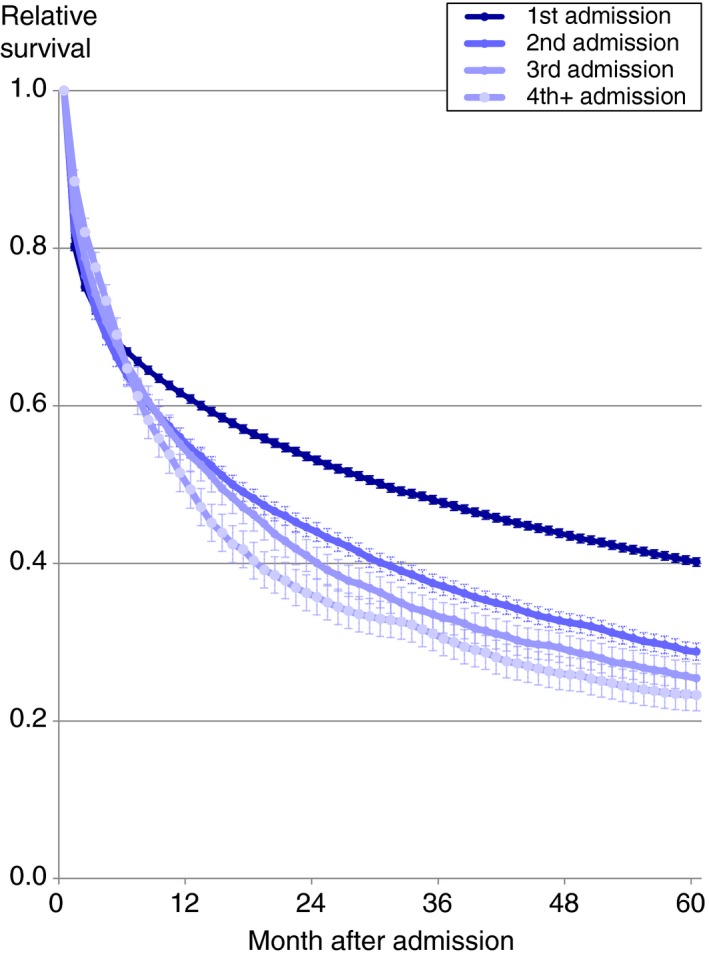
Relative survival up to 5 y following unscheduled admissions for severe liver disease in England and Wales according to the numbers of admissions. Shaded areas represent 95% CIs. Numbers of cases are as follows: 1st admission (n = 33 726), 2nd admissions (7047), 3rd admissions (2391), 4th+ admissions (1788)

Figure 4 shows relative survival according to the numbers of admissions for severe liver disease. At 5 years, relative survival was significantly and substantially better among patients during a first admission for severe liver disease (40.2%) compared with a second admission (28.8%) a 3rd (25.4%) or a 4th further admission (23.3%).

## 4 DISCUSSION

The study shows a very poor overall prognosis following unscheduled hospital admissions for severe liver disease. Mortality was increased by more than 100‐fold in the short term and remained highly elevated throughout 5 years of follow‐up. Mortality was most extremely elevated from liver disease, viral hepatitis and varices. Early 60‐day mortality was substantially lower for patients seen by consultant hepatologists or gastroenterologists. Both early and late mortality were reduced for patients admitted to specialist transplant centres and larger hospitals, those who received liver transplantation, or were resident in London. Early mortality was higher for patients admitted in winter and autumn months, while late mortality was increased with higher levels of patient social deprivation.

Our findings of high early mortality and very poor long‐term prognosis for people hospitalised with severe liver disease are concordant with the limited European literature on this subject. For example, our relative survival at 5 years of 55% for alcoholic hepatitis and 32% for alcoholic liver cirrhosis across England and Wales compares with 53% and 35%, respectively, from a study from Denmark during 2006 to 2011,^21^ and 46% and 29% across Finland from 1996‐2012.^22^ Our figure of 32% for alcoholic liver cirrhosis is almost identical to 33% reported across England from 1998‐2009.^20^^.^

Long‐term prognosis following admissions for alcoholic liver disease is known to be very poor, largely since many cases present with decompensated liver disease when first seen, and subsequent abstinence rates can be low.^8^, ^28^ Although early mortality was much higher for (non‐alcoholic) hepatic failure than for alcoholic liver disease, by 6 months most of the excess mortality for hepatic failure had arisen, whereas longer term survival for alcoholic liver disease continued to deteriorate much more sharply. Of the three main categories of alcoholic liver disease investigated, both early and late mortality were poorest for alcoholic liver failure, reflecting end‐stage liver disease in many cases. Although early mortality for alcoholic hepatitis aetiology was high, longer term prognosis was relatively good. Also, it has been reported that a sub‐diagnosis of alcoholic hepatitis improves survival in patients with compensated alcoholic liver cirrhosis.^13^ Nonetheless, especially in studies based on national administrative inpatient data, there would be variations in definitions and case detection, and also some misclassification of aetiologies. For example, the high SMR from alcoholic liver disease mortality (as the underlying cause of death) for patients admitted with hepatic failure suggests misclassification in some cases. Both alcoholic hepatitis and alcoholic liver failure are more likely to present with more detectable symptoms than compensated alcoholic liver cirrhosis. Without a biopsy, it can also be difficult to distinguish cases of alcoholic hepatitis from decompensated cirrhosis due to alcohol.

Mortality for both alcoholic liver disease and hepatic failure at 5 years was elevated most extremely (>100 and up to 1068‐fold increased risk) from liver disease, viral hepatitis and varices; followed by liver cancer, accidental poisoning, alcohol misuse disorders and, for alcoholic liver disease admissions, oesophageal ulcer, chronic pancreatitis, septicaemia and accidental falls. These findings are similar to those from a large national study of alcoholic liver disease across Finland which reported slightly worse prognosis overall.^22^ For example, our cause‐specific 5‐year SMRs and those reported from Finland are respectively: all causes of death (England and Wales, SMR = 16.7; Finland, 19.9), injury and poisoning (9.7; 11.1), accidental falls (18.9; 20.2), suicide (4.0; 5.0), homicide (3.9; 13.0), circulatory diseases (4.0; 6.1), respiratory diseases (5.2; 7.9), infectious diseases (23.8; 21.0), all cancers (2.1; 6.8) and liver cancer (30.8; 79.0). Our finding of low and/or non‐significant SMRs from non‐liver cancers and from other causes of death that are usually associated with older age groups is partly because of the very high mortality in people with severe liver disease that occurs prematurely in younger and middle age groups. Although acute viral hepatitis infections (A, B, C and E) cause mortality, the extremely high SMR for viral hepatitis is probably attributable more to cirrhosis arising from chronic hepatitis B or C infections.

Both early and late mortality were greatly reduced for patients admitted to specialist transplant centres or resident in London and early mortality was also substantially lower for patients seen by consultant hepatologists or gastroenterologists compared with other specialties. As reported previously,^1^, ^2^, ^3^, ^4^ there is both a shortfall and a higher concentration of transplant centres and hepatology resources in London than in other regions of England and Wales, while liver disease care has been shown to be inadequate in many non‐specialist centres.^2^, ^3^ Consequently, mortality rates vary considerably across hospitals.^2^ Our findings provide further strong evidence that access to specialist resources and expertise improves prognosis.

The finding of lower mortality in largest compared with smaller or non‐specialist hospitals, which is consistent with findings from the Lancet Commission into liver disease,^2^ would also reflect differences in access to expertise, experience and services, relating to hospital size. However, patients who were transferred to transplant centres from elsewhere often had better prognosis (with proportionately more cases of hepatic failure and fewer of alcoholic liver failure), suggesting that there may be selection of cases for transfer based on prognosis. Both early and late mortality were also greatly reduced in patients who received liver transplantation and this reduction widened with longer term follow‐up.

As expected, both early and late mortality were greatly increased in patients seen by critical care specialists. The highest mortality in winter months may also be linked to access as resources become more strained in winter due to increases in admissions for seasonal illnesses, although it may also reflect seasonal variation in alcohol consumption.

We found that late but not early mortality was significantly worse among patients with higher levels of social deprivation and also in Wales compared with England overall. This would reflect more general social inequalities in health,^29^, ^30^ and have previously been reported for severe liver disease,^16^, ^28^ with increases in equalities over the longer term rather than soon after hospital admission and treatment.

### 4.1 Strengths and limitations

Major strengths are that this study is national, one of the largest investigations on prognosis following admission for severe liver disease and it provides new evidence on factors that influence both early and late mortality. The study uses systematic validated record linkage methodology that has been used extensively in previous publications and it covers more than 70 000 admissions for severe liver disease and over 20 000 subsequent deaths. Importantly for confirmatory purposes, it is based on independently collected but similar information sources covering two different populations. The inpatient data sources are based on public hospitals, but these would account for almost all unscheduled admissions for severe liver disease in the two populations.

Limitations are that the national administrative inpatient data used in this study lack detailed information about disease history, disease severity, any therapeutic treatments and also alcohol consumption. Although the study cohorts are based solely on each patient's first admission for severe liver disease during the study period, the information sources do not provide adequate details of how they relate to any possible long‐term previous history of liver disease. We assessed patients who received liver transplants during their study admissions, but were not able to identify from our data patients who subsequently received transplants electively. The principal diagnosis used to determine admissions for severe liver disease is also not accurate in all cases,^31^ while our investigations of the subtypes and aetiologies of severe liver disease and the subsequent causes of deaths are also constrained by the limitations of the ICD coding inherent in national administrative data.

In administrative inpatient data, the consultant specialty managing and treating patients is available only for the first and last episodes of the admission, and the specialties classified do not distinguish hepatology separately from gastroenterology. However, this should still have enabled ascertainment of most of the patients who were managed by hepatologists, gastroenterologists or critical care specialists. Late 5‐year mortality would also be affected by cohort attrition, mainly through population emigration. However, emigration from England and Wales was less than 2.8% per annum during the study period,^32^, ^33^ and it would probably be substantially lower among people with severe liver disease than among the general population. For these reasons, and also since our findings are similar to those from previous cohort studies,^20^, ^21^, ^22^ cohort attrition should be small.

In summary, the study shows a very poor prognosis for people admitted unscheduled for severe liver disease, and several factors that are strongly associated with survival both in the short and long term. In the longer term, prognosis for (non‐alcoholic) hepatic failure is considerably better than for alcoholic liver disease. The study suggests that better access to expertise and specialist services improves survival, both in the short and long term.

## ETHICAL APPROVAL

Ethical approval for the study was not required as it is based on fully anonymised data. Study approval was obtained from the relevant Information Governance Review Panel (IGRP), membership of which includes the National Research Ethics Service, the British Medical Association Ethics Advisor, Caldicott Guardians and members of the public.

## AUTHORSHIP

*Guarantor of the article*: Stephen E. Roberts and Ronan A. Lyons.

*Author contributions*: SER, JGW and JB designed the study; SER reviewed the literature and undertook the analyses; SER wrote the first drafts of the manuscript; SER, JGW, JB, AJ, DN and RAL interpreted the study findings and edited or contributed to subsequent drafts. All authors approved the final version of the article, including the authorship list.

## APPENDIX A

**Table A1:** ICD‐10 codes used for the patient co‐morbidities

Ischaemic heart disease (120‐I25)
Other cardiovascular diseases (I00‐I15, I26‐I52)
Cerebrovascular disease (I60‐I69)
Other circulatory diseases (I70‐I99)
Malignancies (C00‐C97)
COPD (J40‐J44)
Asthma (J45, J46)
Diabetes (E10‐E14)
Renal failure (N17‐N19)
Dementia (F00‐F03, F05.1, G30)
